# Integrating Paper Chromatography with Electrochemical Detection for the Trace Analysis of TNT in Soil

**DOI:** 10.3390/s150717048

**Published:** 2015-07-14

**Authors:** Patrick Ryan, Daniel Zabetakis, David A. Stenger, Scott A. Trammell

**Affiliations:** 1 Science and Engineering Apprenticeship Program, Naval Research Laboratory, Washington, DC 20375, USA; E-Mail: patrick_ryan_@outlook.com; 2 Center for Bio/Molecular Science and Engineering, Naval Research Laboratory, Washington, DC 20375, USA; E-Mails: daniel.zabetakis@nrl.navy.mil (D.Z.); david.stenger@nrl.navy.mil (D.A.S.)

**Keywords:** TNT, paper chromatography, electrochemical detection

## Abstract

We report on the development of an electrochemical probe for the trace analysis of 2,4,6-trinitrotoluene (TNT) in soil samples. The probe is a combination of graphite electrodes, filter paper, with ethylene glycol and choline chloride as the solvent/electrolyte. Square wave chromatovoltammograms show the probes have a sensitivity for TNT of 0.75 nA/ng and a limit of detection of 100 ng. In addition, by taking advantage of the inherent paper chromatography step, TNT can be separated in both time and cathodic peak potential from 4-amino-dinitrotolene co-spotted on the probe or in soil samples with the presence of methyl parathion as a possible interferent.

## 1. Introduction

We have recently implemented a program to develop and demonstrate inexpensive and miniaturized electrochemical sensors for the detection of explosives in the environment. The goal is to enable autonomous devices to carry out analytical techniques without the involvement of trained or skilled personnel. To that end it would be advantageous to combine chromatography with electrochemistry to allow for the separation of analytes and the removal of interferents from environmental samples. The advent of this technology could facilitate the automatic extraction, collection and preparation of analytical samples.

In this manuscript, we report on the development of a paper-based electrochemical probe for the trace analysis of TNT in soil samples. Paper-based devices have several advantages including active transport and chromatographic separation for development of inexpensive sensors in the field. Recent examples in the analytical literature include electrochemical paper based biosensors for the detection of glucose, lactate, and uric acid in biological samples using enzyme based reactions and for heavy-metal ions detection in aqueous solutions [1,2,3,4,5,6,7,8]. We believe explosives detection in the field can also take advantage of the benefits provided by a paper electrochemical device by integrating chromatography with electrochemical detection.

Our probe is a combination of graphite electrodes, filter paper, with ethylene glycol and choline chloride as a deep eutectic mixture solvent/electrolyte. Most electrochemical detection schemes are based on buffered water using simple salts as electrolytes. Room temperature ionic liquids, however, are gaining acceptance as solvents for electroanalytical techniques due to their low vapor pressure, high stability and large voltage window [9]. In addition to room temperature ionic liquids, deep eutectic solvents exhibit similar physical and chemical properties of ionic liquids at a lower cost [10]. In our case, we use a deep eutectic mixture of a 2:1 molar ratio of ethylene glycol and choline chloride. The mixture has a dual function as a solvent and as the electrolyte. The paper-based probes provide both solvent transport for the extraction of the sample from the soil and a chromatography step to separate analytes. These initial results show that the concept of combined chromatography and electrochemistry is a viable approach to developing autonomous explosive detectors.

## 2. Experimental Section 

### 2.1. Materials

Gold films on glass were purchased from Evaporated Metal Films. Mechanical pencil lead (0.7 mm Pentel 50-B) was used as a graphite electrode. Silver/silver chloride ink was purchased from Ercon (catalog # E2414) and used to coat the graphite electrode to construct an Ag/AgCl reference electrode. Ethylene glycol, choline chloride, sandy soil certified reference material, methyl parathion (MeP) and the 2,4,6-trinitrotoluene (TNT) and 4-amino-2,6-dinitrotoluene (4-Am-DNT) standards were purchased from Sigma-Aldrich. Sheet paper was Whatman^®^ 3MM blotting paper.

### 2.2. Sample Preparations

Soil samples were prepared by placing a desired volume of the corresponding stock solution of TNT and/or MeP in 200 µL of methanol. One gram of sandy soil was added to the resulting methanol solution, mixed using a vortex mixer and then place in a vacuum for 3 h to remove the methanol.

### 2.3. Measurements

**Figure 1:**
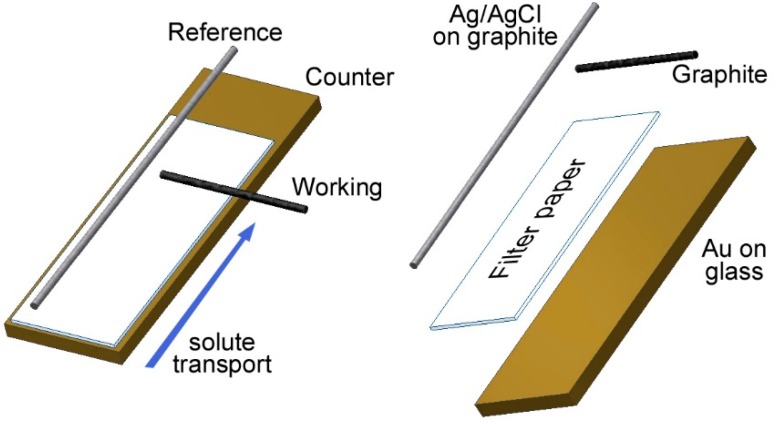
A schematic of our paper-based electrochemical probe.

Electrochemical measurements were performed using a potentiostat model #440 from CH Instruments. The electrolyte for the electrochemical experiments consisted of a deep eutectic mixture of a 2:1 molar ratio of ethylene glycol and choline chloride [11]. To construct the probe, a horizontally-oriented graphite working electrode, and a vertically-oriented reference electrode were clipped to a 2 × 1 cm strip of Whatman filter paper mounted on a counter electrode formed from a 2.54 × 1 cm gold film on glass. A schematic is shown in Figure 1. Square wave voltammetry was measured from 0 to −1 V *vs.* Ag/AgCl at 60 Hz with an amplitude of 25 mV. To correct for the sloping background in the square wave voltammograms, a 5 factor polynomial was fit to each voltammogram on each side of the base of the TNT peak by a least-squares minimization routine using Excel Solver and subtracted from each voltammogram.

### 2.4. Paper Probe Characterization

To characterize the response of the paper-based probe for TNT, the mounted filter paper strip extended 1 cm beyond the end of the gold-plated counter electrode with the working electrode positioned 2 cm from the bottom of the filter paper. The TNT was deposited directly onto the one-centimeter segment of filter paper protracting past the counter electrode from the acetonitrile standard solution and allowed to dry. After each filter paper strip was prepared, 200 µL of the ethylene glycol/choline chloride mixture was placed onto a Fluoroware^®^ tray and the edge of the paper probe was lowered in the solution. The ethylene glycol/choline chloride mixture was allowed to move up the filter paper to the working electrode at which time repetitive runs of square wave voltammetry were initiated.

### 2.5. Soil Sample Analysis

To characterize the response of the paper-based probe for soil samples containing trace amounts of TNT, and MeP, the working electrode was positioned 2 cm from the bottom of the filter paper. One gram of soil sample was placed on a Fluoroware^®^ tray and injected with 200 µL of the ethylene glycol/choline chloride mixture and allowed to equilibrate for 30 s. The bottom edge of the probe was then placed into the wet soil sample at which time repetitive scans of square wave voltammetry was initiated as the ethylene glycol/choline chloride mixture moved toward the working electrode.

## 3. Results and Discussion

### 3.1. Design of the Paper Probe

A schematic of the paper probe is shown in Figure 1 which consisted of a gold film counter electrode on a glass slide and a working and reference electrode clipped on top with a strip of filter paper fitted in-between. We found that pencil lead could be easily positioned on the probe and was a convenient graphite electrode for both working electrode and for the base of the construction of the Ag/AgCl reference electrode [12]. The paper probes were easy to construct, highly responsive for the reduction of TNT and gave reproducible results.

We chose the deep eutectic mixture of ethylene glycol/choline chloride as the solvent/electrolyte since ethylene glycol is slow to evaporate and has a large potential voltage widow for electrochemical detection. For example, at carbon electrodes in ethylene glycol/choline chloride-saturated air, the potential voltage window for ethylene glycol has a range down to −1.7 V *vs.* Ag/AgCl which is ideal for cathodic electrochemistry to detect nitro-aromatics. The ethylene glycol also enables extraction of the analyte from soil, and the filter paper provides both support for the solvent and transport for the chromatographic removal of interferences. To stabilize the mixture, 0.5% (v/v) water was added to the reported formulation [11] in which we note that choline chloride crystalized with extended storage.

For the electrochemical analysis we chose square wave voltammetry which has been commonly used for the detection of nitro-aromatics [13,14,15,16]. In square wave voltammetry, a potential staircase is overlaid on the voltage ramp as the voltage is swept in the desired range at the working electrode. The reduction (or oxidation) of the analyte is then measured as the voltage approaches its formal redox potential. The current is measured at different points of the potential waveform to minimize capacitive charging at the solution-electrode interface. Since non-faradaic current decays faster than faradaic current, a square wave voltammogram can be generated by taking the difference of current between the points subtracting out the capacitive charging current thus making the detection limits typically better then cyclic voltammetry [17].

### 3.2. Square Wave Chromato-Voltammograms

**Figure 2:**
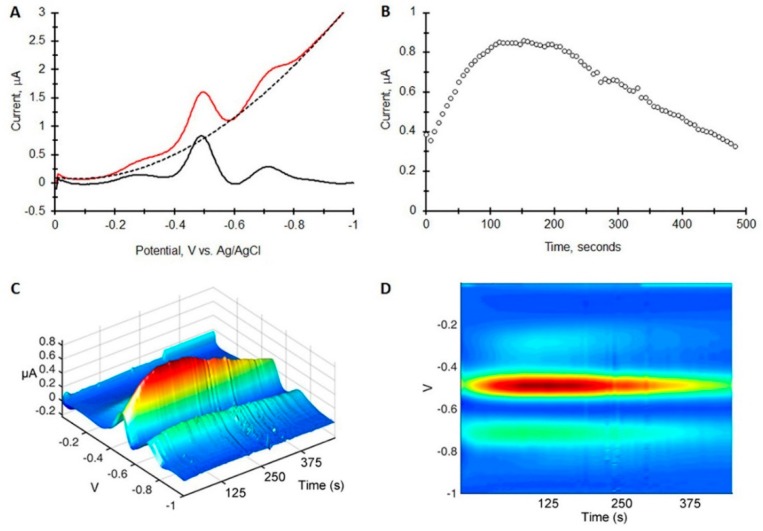
Square wave chromato-voltammogram of 1 µg of TNT on a paper-based electrochemical probe with its edge placed in 200 µL of an ethylene glycol/choline chloride 2:1 molar mixture. Square wave parameters are amplitude = 25 mV, frequency = 60 Hz. (**A**) A square wave voltammogram at the peak maximum in time showing the polynomial background subtraction (dotted line); (**B**) Current measured at I_pc_ *vs.* scan number; (**C**,**D**) 3 dimensional representations of the same square wave chromato-voltammogram.

To characterize the response of the paper probe for TNT detection, square wave chromatovoltammograms were generated by spotting 100 ng to 5000 ng of TNT on the paper, 2 cm below the working electrode and placing the edge of the paper into 200 µL of ethylene glycol/choline chloride mixture. The ethylene glycol/choline chloride mixture was transported up the paper toward the working electrode at which time the electrochemical scans were initiated. A representative data set is shown in Figure 2. To correct for the sloping background in the square wave voltammograms, a 5 factor polynomial was subtracted from each voltammogram (Figure 2A). The resulting chromatovoltammogram is shown in Figure 2B plotting the peak maximum at −0.5 V *vs.* Ag/AgCl *vs.* time. 3D plots are shown in Figure 2C,D. From the data, it is apparent that the TNT reaches the working electrode generating a signal near the solvent front. The signal then reaches maximum near 150 s and decays with a tail extending beyond 500 s.

**Figure 3:**
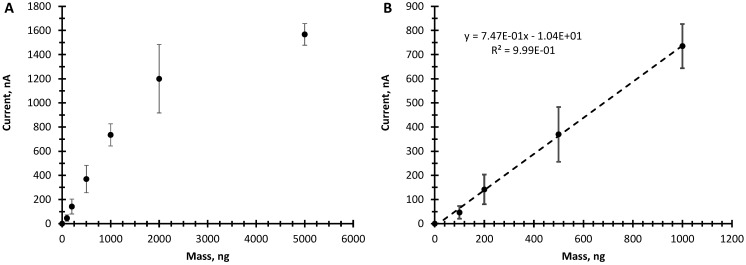
(**A**) Peak maximum in time *vs.* mass of TNT placed on the paper-probe from 100 to 5000 ng; (**B**) Current *vs.* mass of TNT in the linear range of 100 to 1000 ng.

In Figure 3, the amplitude of the peak at 100 s is plot *vs.* mass of TNT. The linear range is between 100 to 1000 ng with a sensitivity of 0.75 nA/ng and a limit of detection (LOD) of ~100 ng. The LOD was calculated by 3 times the standard deviation of the lowest concentration measured divided by the slope of the regression curve [18]. Beyond 1000 ng the response of the paper probe begins to saturate. The nature of the saturation most likely is caused by various parameter interactions including the slow diffusion coefficient of the TNT in the ethylene glycol, the mass transport of TNT in the ethylene glycol as it is wicked up to the working electrode and rate of electron transfer at the working electrode.

### 3.3. Integrating Paper Chromatography

**Figure 4:**
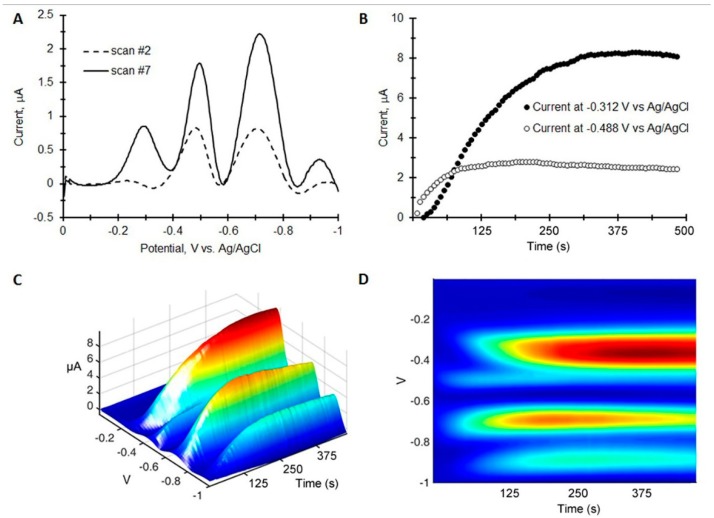
Separation of 5 µg of 4-Am-DNT and TNT placed on the paper-based electrochemical microelectrode (**A**) Square wave voltammograms at different time points; (**B**) Time traces at two different potentials; (**C**,**D**) 3D square wave chromatovoltammograms. Parameters: Amplitude = 25 mV, Frequency = 60 Hz.

We chose to examine a 1:1 mixture of TNT and 4-Am-DNT to characterize the ability of the paper to separate similar compounds. 4-Am-DNT is a decomposition product of TNT and is found in soil of contaminated sites. In the experiment, 1000 ng of TNT and 4-Am-DNT were co-spotted on the paper and a chromatovoltammogram was generated as before. The resulting chromatovoltammograms are shown in Figure 4B plotting the peak maximum at −0.3 V and −0.5 V *vs.* Ag/AgCl *vs.* time. A 3D plot is shown in Figure 4C,D.

Two observations are clear: the 4-Am-DNT has a larger retention time with the peak maximum at 380 s for 4-Am-DNT, and the peak amplitude for 4-Am-DNT is larger suggesting that 4-Am-DNT generates a stronger signal as compared to TNT. The 3D plots show that TNT and 4-Am-DNT can be separated both in time and in cathodic peak potential.

The paper probes also perform well in the extraction and separation of analytes and interferences in soil. To demonstrate this, we chose to analyze a mixture of TNT and methyl parathion in sandy soil. Methyl parathion is a broad spectrum organothiophosphate insecticide and hazardous pollutant and is used for the treatment of agricultural crops [19]. The compound contains a nitro-aromatic ring which could lead to interference and signal distortion when detecting TNT in soil near agricultural areas.

**Figure 5:**
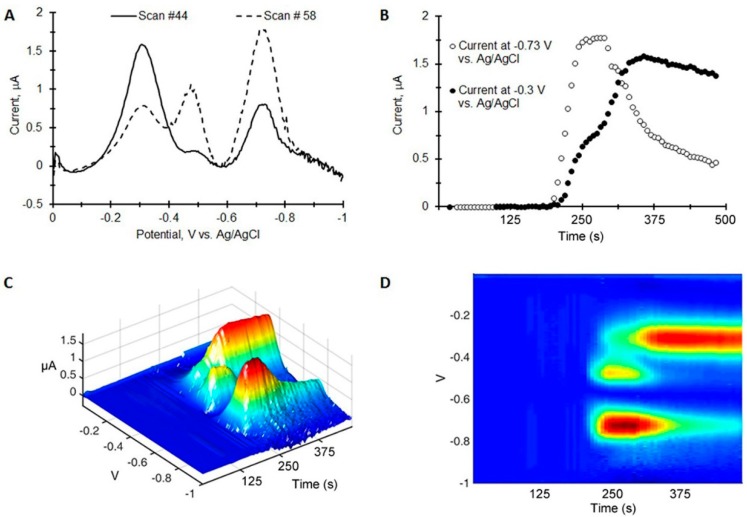
Separation of MeP and TNT in sandy soil. (**A**) Square wave voltammograms at different time points; (**B**) Time traces at two different potentials; (**C**,**D**) 3D square wave chromato-voltammograms. Parameters: Amplitude = 25 mV, Frequency = 60 Hz.

In the experiment, the ethylene glycol/choline chloride mixture was added to a contaminated soil sample and after an equilibration time of 30 s, the edge of the probe was place into the wet soil. The ethylene glycol/choline chloride mixture with dissolved analyte was transported up to the working electrode. Figure 5 shows the resulting chromatovoltammograms from a 1 g soil sample containing 1000 ng of TNT and MeP. In this case, the electrochemical testing was started immediately after placement into the soil. At 200 s the solvent front reached the working electrode followed by the signal for TNT at 273 s. At 380 s the MeP signal at −0.3 V *vs.* Ag/AgCl reaches a maximum at the electrode. The presence of the MeP seems to block the TNT electrochemistry. The thiophosphate group of the MeP most likely is the cause due to its longer retention time and electrode blocking characteristics. Nevertheless, it is clear that the two analytes can be individually detected in the soil sample both in time and cathodic peak potential by exploiting the chromatographic and electrochemical aspects of the paper probe.

## 4. Conclusions

We have shown that a simple paper-based electrochemical probe can be used for the analysis of TNT in soil. The combination of square wave voltammetry and the chromatographic nature of filter paper using ethylene glycol/choline chloride as a solvent/electrolyte generate 3D chromatovoltammograms that can be used to separate different nitro-aromatics in time and applied potential at a working graphite electrode. In addition, the technique possesses a broad spectrum of applications because its pair of separation mechanisms provides meaningful, rapid results which avoid the harmful effects of interferences, an inevitable obstacle to implementation. Possible purposes include the enabling of autonomous systems to carry out the remote electrochemical detection of explosives, as well as assisting manned missions in on-site, immediate explosive sensing, principally for the Department of Defense.

## References

[1] Santhiago M., Kubota L.T. (2013). A new approach for paper-based analytical devices with electrochemical detection based on graphite pencil electrodes. Sens. Actuators B Chem..

[2] Santhiago M., Henry C.S., Kubota L.T. (2014). Low cost, simple three dimensional electrochemical paper-based analytical device for determination of p-nitrophenol. Electrochim. Acta.

[3] Nery E.W., Kubota L.T. (2013). Sensing approaches on paper-based devices: A review. Anal. Bioanal. Chem..

[4] Liana D.D., Raguse B., Gooding J.J., Chow E. (2012). Recent Advances in Paper-Based Sensors. Sensors.

[5] Liu B.W., Du D., Hua X., Yu X.Y., Lin Y.H. (2014). Paper-Based Electrochemical Biosensors: From Test Strips to Paper-Based Microfluidics. Electroanalysis.

[6] Dungchai W., Chailapakul O., Henry C.S. (2009). Electrochemical Detection for Paper-Based Microfluidics. Anal. Chem..

[7] Carvalhal R.F., Kfouri M.S., Piazetta M.H.D., Gobbi A.L., Kubota L.T. (2010). Electrochemical Detection in a Paper-Based Separation Device. Anal. Chem..

[8] Nie Z., Nijhuis C.A., Gong J., Chen X., Kumachev A., Martinez A.W., Narovlyansky M., Whitesides G.M. (2010). Electrochemical sensing in paper-based microfluidic devices. Lab Chip.

[9] Ho T.D., Zhang C., Hantao L.W., Anderson J.L. (2013). Ionic Liquids in Analytical Chemistry: Fundamentals, Advances, and Perspectives. Anal. Chem..

[10] Zhang Q.H., Vigier K.D., Royer S., Jerome F. (2012). Deep eutectic solvents: Syntheses, properties and applications. Chem. Soc. Rev..

[11] Shahbaz K., Mjalli F.S., Hashim M.A., ALNashef I.M. (2010). Using Deep Eutectic Solvents for the Removal of Glycerol from Palm Oil-Based Biodiesel. J. Appl. Sci..

[12] Dossi N., Toniolo R., Terzi F., Impellizzieri F., Bontempelli G. (2014). Pencil leads doped with electrochemically deposited Ag and AgCl for drawing reference electrodes on paper-based electrochemical devices. Electrochim. Acta.

[13] Galik M., O’Mahony A.M., Wang J. (2011). Cyclic and Square-Wave Voltammetric Signatures of Nitro-Containing Explosives. Electroanalysis.

[14] Johnson B., Nasir M., Siefert R., Leska I., Erickson J., Charles P., Melde B., Taft J. (2014). Electrochemical Detection with Preconcentration: Nitroenergetic Contaminants. Chemosensors.

[15] Wang J. (2007). Electrochemical Sensing of Explosives. Electroanalysis.

[16] Trammell S.A., Zabetakis D., Moore M., Verbarg J., Stenger D.A. (2014). Square wave voltammetry of TNT at gold electrodes modified with self-assembled monolayers containing aromatic structures. PLoS ONE.

[17] Bard A.J., Faulkner L.R. (2001). Electrochemical Methods: Fundamentals and Applications.

[18] Harris D.C. (2007). Quantitative Chemical Analysis.

[19] Tabassum N., Rafique U., Balkhair K.S., Ashraf M.A. (2014). Chemodynamics of Methyl Parathion and Ethyl Parathion: Adsorption Models for Sustainable Agriculture. Biomed. Res. Int..

